# Muscle Sympathetic Nerve Activity Is Related to a Surrogate Marker of Endothelial Function in Healthy Individuals

**DOI:** 10.1371/journal.pone.0009257

**Published:** 2010-02-17

**Authors:** Yrsa Bergmann Sverrisdóttir, Linda Marie Jansson, Ulrika Hägg, Li-Ming Gan

**Affiliations:** Institute of Neuroscience and Physiology, Department of Physiology, Sahlgrenska Academy, University of Gothenburg, Gothenburg, Sweden; University of British Columbia, Canada

## Abstract

**Background:**

Evidence from animal studies indicates the importance of an interaction between the sympathetic nervous system and the endothelium for cardiovascular regulation. However the interaction between these two systems remains largely unexplored in humans. The aim of this study was to investigate whether directly recorded sympathetic vasoconstrictor outflow is related to a surrogate marker of endothelial function in healthy individuals.

**Methods and Results:**

In 10 healthy normotensive subjects (3 f/7 m), (age 37±11 yrs), (BMI 24±3 kg/m^2^) direct recordings of sympathetic action potentials to the muscle vascular bed (MSNA) were performed and endothelial function estimated with the Reactive Hyperaemia- Peripheral Arterial Tonometry (RH-PAT) technique. Blood samples were taken and time spent on leisure-time physical activities was estimated. In all subjects the rate between resting flow and the maximum flow, the Reactive Hyperemic index (RH-PAT index), was within the normal range (1,9–3,3) and MSNA was as expected for age and gender (13–44 burst/minute). RH-PAT index was inversely related to MSNA (r = −0.8, p = 0.005). RH-PAT index and MSNA were reciprocally related to time (h/week) spent on physical activity (p = 0.005 and p = 0.006 respectively) and platelet concentration (PLT) (p = 0.02 and p = 0.004 respectively).

**Conclusions:**

Our results show that sympathetic nerve activity is related to a surrogate marker of endothelial function in healthy normotensive individuals, indicating that sympathetic outflow may be modulated by changes in endothelial function. In this study time spent on physical activity is identified as a predictor of sympathetic nerve activity and endothelial function in a group of healthy individuals. The results are of importance in understanding mechanisms underlying sympathetic activation in conditions associated with endothelial dysfunction and emphasise the importance of a daily exercise routine for maintenance of cardiovascular health.

## Introduction

While the sympathetic nervous system plays a central role in the regulation of the cardiovascular system the endothelium plays a key role in the local regulation of peripheral vascular tone and structure. As both systems play a crucial role in short- and long term adjustments of the cardiovascular system both are heavily involved in the development and prognosis of cardiovascular events and disease [1] [2], [3].

Evidence from animal studies indicates the importance of an interaction between the sympathetic nervous system and the endothelium for cardiovascular regulation [4].

Though such data from human studies is limited, independent observations in previous studies may suggest a reciprocal relationship between endothelial function and the activity of the sympathetic nervous system. An acute increase in sympathetic activity has been shown to cause a decrease in endothelial function [5]. Men tend to have higher sympathetic outflow than women in reproductive age, who in turn have higher endothelial nitric oxide (NO) production [6]. Sympathetic nerve activity is augmented in the early morning before waking, while endothelial function has been reported to attenuate [7], [8]. Beta adrenergic-blockade treatment aimed at reducing sympathetic nervous activity, raises endothelial function [9]. Aging is associated with increased sympathetic outflow [10] and decreased endothelial function [11].

Though these observations are not evidence of a causal relationship, the number of independent observations makes such a hypothesis feasible. Furthermore, hypertension, insulin resistance, adult onset growth hormone (GH)-deficiency, polycystic ovary syndrome (PCOS) and congestive heart failure (CHF), are conditions coupled with severely decreased endothelial function [12], [13], [14] and greatly increased sympathetic nerve activity [1], [15], [16], [17]. In these conditions, physical activity thought to be protective against the development of vascular disease, has been shown to have beneficial effects on both sympathetic outflow and endothelial function [18], [19], [20], [21], [22], [23].

Against this background, the aim of this study was to investigate if directly recorded sympathetic vasoconstrictor outflow is related to a surrogate marker of endothelial function, the RH-PAT index, in physically active, normotensive, healthy individuals.

## Results

Table 1 summarises the basic characteristics and laboratory values of the study group. MSNA was as expected, lower in females than males (p = 0.01) and increased with age (p = 0.05) in this cohort of subjects [34] while the RH-PAT index was not related to age and gender.

**Table 1:** Basic characteristics of the 10 (7 M/3 F) subjects shown in mean, SD and range.

Variable	Mean	SD	Range
Age, years	37	11.3	24–61
Body mass index, kg/m2	24	2.7	20–30
Heart rate, beats/min	59	12	40–80
Systolic BP, mmHg	121	7.6	109–133
Diastolic BP, mmHg	70	6.5	62–81
Mean Arterial Pressure, mmHg	86	5.5	77–95
**Microneurography and RH-PAT**			
MSNA, bursts/min	33	10.5	13–44
MSNA, bursts/100 heart beats	57	17.6	28–88
Reactive Hyperemic Index (%)	2.3	0.5	1.58–3.33
**Blood samples**			
Triglycerides (mmol/L)	0.98	0.4	0.4–1.55
Apolipoprotein A (g/L)	1.26	0.15	1.07–1.55
Apolipoprotein B (g/L)	0.86	0.23	0.5–1.22
Cholesterol (mmol/L)	4.8	1.11	3.4–6.2
Low Density Lipoprotein (mmol/L)	99	50.4	0.00–175
High Density Lipoprotein (mmol/L)	1.48	0.28	1.0–1.84
Insulin-like Growth Factor-I (ug/L)	209	69.8	130–327
Epidermal growth factor (pg/ml)	57	24.7	30.5–118.8
Platelet concentration (×10 g/L)	227	51.3	146–309

### MSNA, RH-PAT Index and Physical Activity

**Figure 1:**
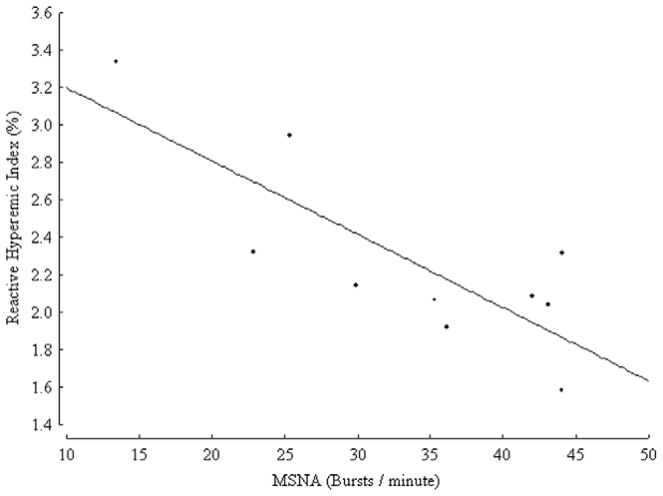
Muscle sympathetic nerve activity (MSNA) expressed as burst frequency (bursts/minute) and reactive hyperemic index (%) in 10 healthy controls, r = −0.8, p = 0.005.

**Figure 2:**
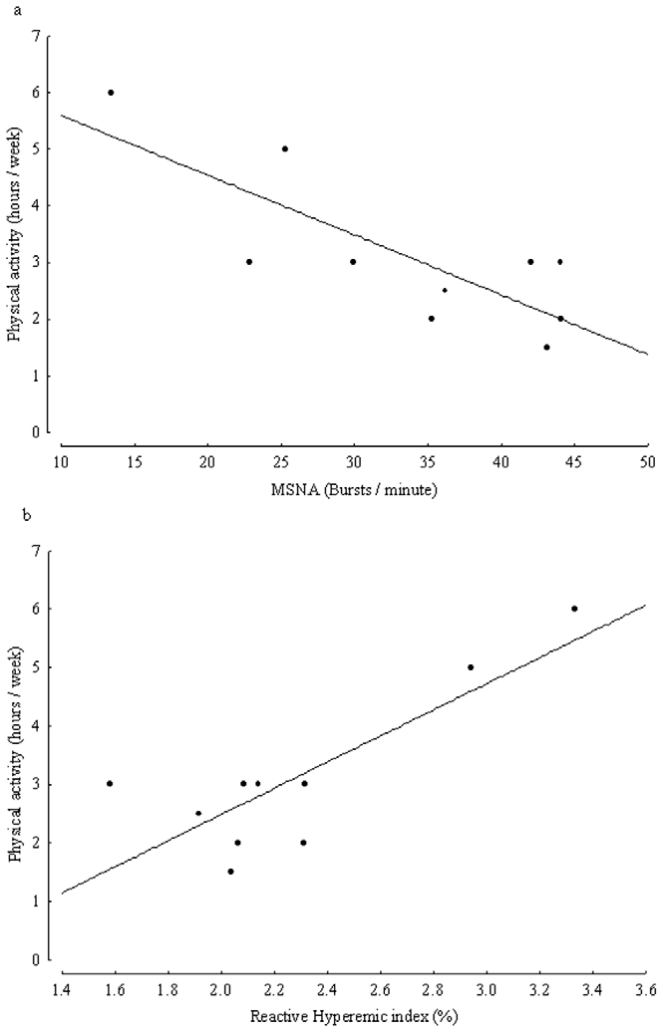
Physical activity expressed as hours per week and a) muscle sympathetic nerve activity (MSNA) expressed as burst frequency (bursts/minute) and b) reactive hyperaemic index (%) in 10 healthy controls, (r = −0.79, p = 0.006 and r = 0.83, p = 0.004, respectively).

Table 2 summarises the results of a simple correlation matrix illustrating that MSNA was inversely related to the RH-PAT index (r = −0.8, p = 0.005, figure 1), and time spent on physical activity (h/wk) (r = −0.79, p = 0.006, figure 2a) and positively related to PLT and EGF concentration (p = 0.02 for both). All relationships were independent of age and gender. MSNA was not related to other measured parameters in this study.

**Table 2:** Simple correlation matrix showing associations between Muscle Sympathetic Nerve Activity (MSNA), Reactive Hyperemic Index (RHI), Platelet concentration (PLT), Epidermal growth factor (EGF), Insulin-Like Growth Factor (IGF)-I, and physical activity (PA) for the study group.

	MSNA	RHI	PLT	EGF	IGF-I
**RHI**	−0.80***				
**PLT**	0.73*	−0.85***			
**EGF**	0.75*	−0.45	0.58		
**IGF-I**	−0.50	0.49	−0.45	−0.36	
**PA**	−0.79**	0.83***	−0.72*	−0.06	0.35

*P*-values are indicated as: *p≤0.05; **p≤0.01; ***p≤0.005.

The RH-PAT index was positively related to time spent on physical activity (h/wk) (r = 0.83, p = 0.004, figure 2b) and inversely related to PLT concentration (p = 0.004), but not to other parameters measured.

In addition to being related to MSNA and the PH-PAT index, time spent on physical activity (h/wk) was inversely related to PLT concentration (p = 0.03).

In a multiple linear regression analyses (power = 0.8), time spent on physical activity (h/wk) and PLT concentration were able to explain the relation between MSNA and the RH-PAT index, but physical activity (h/wk) had a stronger impact than PLT concentration (regression weights; −0.3 (p = 0.5) and 0.1 (p = 0.7), respectively).

## Discussion

The importance of an interaction between the sympathetic nervous system and the endothelium for cardiovascular regulation is becoming increasingly evident, but data from human studies is limited.

This study demonstrates that in healthy individuals without any evidence of vascular atherosclerosis, directly recorded sympathetic nerve activity is inversely related to a surrogate marker of endothelial function, the RH-PAT index. Furthermore, self-reported time spent on physical activity assessed in hours per week, was inversely related to MSNA and positively related to RH-PAT index, indicating that the more time spent on physical activity the lower your sympathetic outflow and better your endothelial function. Though correlations can not serve as evidence of a causal relationship, the results are of interest and render for further investigation into underlying mechanisms.

### Sympathetic Nerve Activity, Endothelial Function and Physical Activity

In this study physical activity is identified as an independent predictor of sympathetic nerve activity and endothelial function in a group of healthy individuals. Physical activity has been shown to greatly improve endothelial function in both health and disease [35], [36] and to play a early protective role for cardiovascular health [24]. The effects of physical exercise on sympathetic nerve activity in healthy subjects have however yielded conflicting results, showing both an increase and decrease in resting MSNA [37].

Exercise induces favourable gene expression in endothelial cells, ie. nitric oxide (NO) synthase and extracellular superoxide dismutase (SOD), which improves NO biological activity [38]. Bioavailability of NO has been proposed to be tonically involved in the restraint of central sympathetic outflow [39], [40], [41] which is in line with a previous finding from our group of an inverse relation between plasma nitrate level and sympathetic nerve activity in aging men [16]. Physical fitness improves endothelial function in older men [11] by increasing bioavailability of vascular NO and has beneficial effects on the age-related increase in sympathetic outflow by decreasing sympathetic activity during extended physical exercise [22]. This effect was recently demonstrated by Gao and coworkers [42] to be associated with upregulation of SOD expression in the central nervous system.

Reactive oxygen species (ROS), which decreases the bioavailability of NO, plays a critical role in the development of endothelial dysfunction [43] and has been shown to be involved in activation of the sympathetic nervous system [44]. ROS is chronically elevated in many conditions related to endothelial dysfunction such as, atherosclerosis, type 2 diabetes mellitus, hypertension and cardiac failure [45], [46] which in turn are also associated with increased sympathetic nerve activity. Endothelial dysfunction crucially contributes to the reduced exercise capacity in patients with congestive heart failure, with fundamental impact on morbidity and mortality. Physical exercise has been shown to reduce MSNA in heart failure [18] and to alter HRV in type 2 diabetes[21], an effect proposed to originate from a central effect of training [22]. Furthermore, physical exercise increases NO bioavailability, augments insulin sensitivity and decreases sympathetic nerve activity in insulin resistant subjects [19]. It is possible that in conditions associated with endothelial dysfunction [12], [13], [14] [1], [15], [16] an impairment in the synthesis and/or release of NO may contribute to the sustained sympathetic activation seen in these conditions.

The measurement of digital RH-PAT was recently shown to be a useful test of NO bioavailability and endothelial function in healthy individuals [47]. It may therefore be speculated that the beneficial effects of physical activity on sympathetic outflow and the RH-PAT index in our study, could be related to augmented NO bioavailability [40]. In this study platelet concentration was also able to explain the relation between sympathetic outflow and the RH-PAT index, though its impact was weaker than that of time spent on physical activity. As endothelial derived NO is a major endogenous modulator of platelet function, the relationships seen between platelet concentration, MSNA and RH-PAT index in this study may reflect the extent of NO bioavailability in our subjects and support the notion that NO might be a possible afferent mediator responsible for the relationships.

Sympathetic nerve activity tends to increase [10] and endothelial function to decrease with advancing age [11]. An age-related reduction in the activity of the somatotropic hormonal (GH)/IGF-I) axis may be suggested as a plausible explanation as this axis is involved in the regulation of central sympathetic outflow [48] as well as in endothelial production of NO [49] and stimulation of NO production in vascular smooth muscle cells. In this study serum IGF-I was not significantly related to MSNA or the RH-PAT index and can therefore not explain the relationships observed.

Men tend to have higher sympathetic outflow than women. The underlying mechanisms remain largely unclear [34], but evidence from both human and animal studies indicate that sex hormones may modify the synthesis/bioactivity of NO. Total NO production is greater in women at reproductive age than in men [6] and endothelial NO release is greater in blood vessels of female than male rats [50]. We have recently shown that women with PCOS, the most common endocrine cause of infertility in young women, known to be associated with decreased endothelial function [51] have severely increased sympathetic outflow. The increased sympathetic nerve activity was shown to be due to the excessive testosterone levels distinguishing the condition [17]. In this study, total testosterone level was measured but was not related to sympathetic nerve activity or the RH-PAT index.

In conclusion: the novel finding in this study is that in a group of healthy subjects, sympathetic outflow is related to a surrogate marker of endothelial function, the RH-PAT index, a relationship explained by the number of hours per week spent on physical activity. The results lend support to the hypothesis of an interaction between the sympathetic nervous system and endothelium in cardiovascular regulation and emphasise the importance of a daily exercise routine for maintenance of cardiovascular health.

Though the statistical correlation between MSNA and the RH-PAT index in this study cannot serve as evidence for a causal relationship between the systems, the results are of importance in understanding mechanisms underlying increased sympathetic outflow in conditions associated with decreased endothelial function.

### Limitations of the Study

A limitation to this study is the lack of identifying the specific mechanisms responsible for the observed interesting phenomenon and we can only speculate that a potential afferent candidate may involve nitric oxide. A further limitation is that only healthy, physically active, normotensive individuals were recruited and studied. However, whether the present relationship between sympathetic outflow and endothelial function also is evident in healthy sedentary individuals as well as in conditions associated with endothelial dysfunction awaits future studies.

As the RH-PAT probe is placed on the digit where there are no muscles, it may be argued that the RH-score could reflect skin sympathetic nerve activity (SSNA) rather than MSNA. As SSNA is mainly involved in thermoregulation the RH-index would reflect skin vascular physiology rather than systemic hemodynamics. Interestingly, though skin vascular physiology cannot explain systemic hemodynamics, both skin vasodilatation and flow mediated vasodilatation were recently shown to be markedly attenuated in the early morning hours in healthy subjects [7], [52]. In this study, SSNA was obtained in five out of ten subjects, but no clear relationship was found between this branch of the sympathetic nervous system and the RH-index.

## Methods

### Subjects

Ten healthy non-athletic but physically active subjects (3 females/7 males) with a median age of 35 years (range 24–61 yrs) and a body mass index (BMI) of 23 kg/m^2^ (range 20–30), free from medication and without any former history of cardiovascular disease or diabetes mellitus, were recruited to the study. A written informed consent was attained from all the participants in this study, which was approved by the local ethics committee at Sahlgrenska University Hospital, Gothenburg, Sweden.

### Study Design

On the first visit reactive hyperemia arterial tonometry (RH-PAT) measurements were performed. On the second visit and after an overnight fast blood samples were taken for triglycerides, cholesterol, apolipoprotein A1 and B, epidermal growth factor (EGF), insulin-like growth factor (IGF)-I, total testosterone and Thyrotrophic stimulating hormone (TSH). On the third visit, body height and weight, resting blood pressure and a microneurographic recording of muscle sympathetic nerve activity (MSNA) were performed. Body weight was measured to the nearest 0.1 kg, and body height was measured barefoot to the nearest 0.01 m. The body mass score (BMI) was calculated as body weight in kilograms divided by height in meters squared. Systolic (SBP) and diastolic (DBP) blood pressure was measured using a calibrated sphygmomanometer to the nearest 5 mmHg during 15 minutes of supine rest. Average blood pressure values were derived from 3 consecutive measurements. Mean arterial pressure (MAP) was calculated by the formula MAP  =  DBP + (SBP-DBP)/3.

### Assessment of Physical Activity History

All participants reported the average amount of time spent weekly on high-intensity aerobic training (including jogging, swimming, participation in aerobics, football, etc.) and daily activity (including walking or bicycling to work). Total self-reported physical activity, recently demonstrated to be positively correlated to steady-state *V*· O_2__maxc_ in healthy young individuals [24], was calculated as the sum of time spent weekly on high-intensity aerobic training and daily activity expressed as hours per week (h/wk).

### Biochemical Measurements

All biochemical analyzes were performed using commercially available kits, according to the Manufacturer's protocols. Serum triglycerides (TG), cholesterol, low (LDL)- and high density lipoprotein (HDL)-cholesterol and plasma glucose was measured with enzymathic photometric method, 37°C (TG, Roche/Hitachi, CHOL, Roche/Hitachi, HDL-C 2^nd^ generation, Roche/Hitachi, Roche Diagnostics, GmbH, Mannheim, Germany). The assays were measured on Cobas mira analyzer (Hoffman- La Roche & Co., Basle, Switzerland).

The Apolipoprotein A1 and B concentrations were measured with turbid metric technique, using polyclonal rabbit anti-human antibodies (Q 0496 and Q 0497, Daco Cytomation, Glostrup, Denmark). Epidermal growth factor (EGF) was measured using Randox Protein Chip technology [25]. Serum total testosterone was measured by competitive immunochemistry with chemoluminiscence technology (ADVIA Centaur® TSTO Ready Pack® primary reagens, Bayer Health Care LLC, Tarrytown, NY, US). Serum Insulin-like growth factor (IGF)-1 was measured with immunochemoluminiscence technology (Immulite 2500 IG1, Euro/DPC, UK). Thyroid-stimulating hormone (TSH) was measured with electrochemiluminescence immuno assay (ECLIA), 37°C (TSH Thyrotropin, Roche Diagnostics GmbH, Mannheim, Germany).

### Reactive Hyperemia Arterial Tonometry (RH-PAT)

Endothelial function was estimated by using the Endo-PAT device (Itamar Medical Ltd., Caesarea, Israel), a noninvasive technology that captures a beat-to-beat plethysmographic recording of the finger arterial pulse wave amplitude (PWA) with pneumatic probes, detecting the peripheral arterial tone. Peripheral arterial tonometry (PAT) was assessed in response to reactive hyperemia. Subjects were in a comfortable sitting position with hands at heart level. Digit probes were placed on both index fingers and a continuous recording of pulsatile blood volume response from both hands was initiated. A blood pressure cuff was placed on the study arm, while the contra-lateral arm served as control. The subjects were allowed to rest for fifteen minutes before a five minute occlusion of the brachial artery was performed by inflating the blood pressure cuff to 60 mmHg above systolic pressure. Following the occlusion, the cuff was deflated to allow flow-mediated reactive hyperemia (RH) while the PAT recording was continued. Pulse wave amplitudes were recorded for 5 minutes after the cuff was deflated. As a measure of extent of RH, the RH-PAT index was calculated as the ration of the average amplitude of the PAT signal over a 1 minute time interval starting one minute after cuff deflation divided by the average amplitude of the PAT signal over a 3.5 minute time period before cuff inflation (baseline) [26]. The RH-PAT index values from the study arm are then normalized to the concurrent signal from the contralateral finger to correct for changes in systemic vascular tone. The RH-PAT index was recently shown to serve as a surrogate marker of endothelial dysfunction in children with type 1 diabetes [27].

Since the ITAMAR method is relatively user-independent and all the measurements and calculations were performed in an automatic way by means of the software, the technical reproducibility is high [28].

### Microneurography

Direct recordings of multiunit efferent postganglionic muscle sympathetic nerve activity (MSNA) were obtained with a tungsten microelectrode with a tip diameter of a few microns inserted into a muscle fascicle of the peroneal nerve, posterior to the fibular head. A low impedance reference electrode was inserted subcutaneously a few centimetres away. When a muscle nerve fascicle had been identified, small electrode adjustments were made until a site was found in which spontaneous, pulse-synchronous bursts of neural activity could be recorded. Details of the nerve recording technique and criteria for MSNA have been reported previously [29]. Bursts identified by inspection of the mean voltage neurogram were expressed as burst frequency (bursts per min) and burst incidence (bursts per 100 heartbeats). The nerve recordings were assigned a code and analysed blinded.

During the microneurographic recording, finger arterial blood pressure was measured non-invasively by the volume-clamp method, (Finapress 2300, Ohmeda, Louisville, USA) [30], heart rate was monitored via ECG-chest electrodes and respiration via a strain-gage strapped around the waist.

MSNA consists of baroreceptor reflex controlled vasoconstrictor impulses to the muscle vascular bed, involved in dynamic blood pressure regulation. Although MSNA only represents one subdivision of the sympathetic nervous system, at rest it correlates well with a global measure of sympathetic nerve activity such as total body norepinephrine spillover, and with regional (heart and kidney) norepinephrine spillover [31], [32].

Though MSNA differs between different individuals, it has been shown to have strong intra-individual reproducibility over many years, which makes it possible to monitor long-term changes in MSNA, both in disease and therapeutic interventions [10], [33].

### Statistics

Statistical analysis was performed using STATISTICA 7 for windows (StatSoft, Inc., Tulsa, OK). Results are presented as the mean and SD. Correlations were examined by calculating the Pearson linear correlation coefficient and a multivariate analysis model was used to adjust for age and gender. A power value ≥0.8 was considered acceptable. A p-value ≤0.05 was considered statistically significant.
